# Evaluation of Chemical and Microbiological Quality in 21 Brands of Iranian Bottled Drinking Waters in 2012: A Comparison Study on Label and Real Contents

**DOI:** 10.1155/2013/469590

**Published:** 2013-04-15

**Authors:** M. Moazeni, M. Atefi, A. Ebrahimi, P. Razmjoo, M. Vahid Dastjerdi

**Affiliations:** ^1^Department of Environmental Health Engineering, School of Health, Student Research Center, Isfahan University of Medical Sciences (IUMS), Isfahan, Iran; ^2^Department of Environmental Health Engineering, School of Health, Environment Research Center, Isfahan University of Medical Sciences (IUMS), Isfahan 8174673461, Iran; ^3^Water & Wastewater Laboratory, School of Health, Isfahan University of Medical Sciences (IUMS), Isfahan, Iran

## Abstract

The purpose of this study was to evaluate and compare chemical and microbiological quality of the 21 Iranian bottled drinking waters reported on manufacturer's labeling and standards in 2012. Samples were analyzed for chemical properties K^+^, F^−^, SO_4_^2−^, Cl^−^, Mg^2+^, Ca^2+^, and pH. Total and fecal coliform and heterotrophic plate counts of selected samples were analyzed by MPN and HPC tests, respectively, for three months. Finally, the labeled and real contents of the samples were compared. Potassium and sulfate ions about 43 and 52 percent of studied sample contents had values higher than label amounts, respectively. Ca^2+^, Cl^−^ ions, and pH were about 71, 48, and 67 percent, respectively, less than label values. Total and fecal coliforms had negative results. The mean concentrations and standard deviations for K^+^, Cl^−^, pH, Ca^2+^, Mg^2+^, SO_4_^2−^, and HPC were 1.13 ± 1.06, 16.39 ± 31.97, 6.6 ± 0.7, 28.35 ± 10.34, 86.58 ± 33.21, 24.17 ± 17.30 mg/L, and 16855 ± 25603 cfu/mL, respectively. Thus, there is possibility of microorganisms' growth in favorite conditions in bottled water. It was imperative to assess the public health risks in bottled water in Iran.

## 1. Introduction

Recently, the consumption of bottled mineral waters has increased worldwide [1–3], because people living in developed countries have no suitable water supply at their homes [4]. Water quality can have a major impact on both individuals and communities health [5]. It is very important to human health to ensure the safety of consuming drinking water [6, 7]. Drinking water is important for survival, so that its biological and chemical contamination is a serious problem that may have severe health effects [7]. Human activities increase contamination levels through different point and nonpoint sources, which eventually cause them to be present in our drinking water resources [8]. Increasing pollution in drinking waters amplified the demand for natural mineral waters [5]. Natural mineral waters are defined as a microbiological healthy water originating in an groundwater tables or deposits and emerging from a spring tapped at one or more natural or bore exits with an individual and constant chemical composition [9, 10]. Casanovas-Massana and Blanch described that natural mineral waters are complex environments containing a large variety of autochthonous microbiota [11]. Mineral water is characterized by its purity at source, its content in minerals, trace elements, and other constituents [12]. Falcone-Dias et al. reported that mineral waters have been marketed as ideal for infant formula preparation and nursery drinking water, particularly for the immunosuppressed people [9]. In assessing the quality of drinking waters, consumers rely principally upon their senses with bottled waters being perceived as pure, safe, and of good taste; thus, their consumption is increasing despite the excessively high prices compared to tap water [3, 13]. Bottled mineral waters are relatively large proportion of bottled water (between 40 and 60% globally) [14]. Bottled drinking water has been occasionally related to diarrhea conditions known as traveller's disease. Although the microbial quality levels in processed water are often initially low, bottled mineral water is not a sterile product and can contain naturally occurring bacteria as well as those introduced during manufacturing or consumer handing [13, 15]. Fernanda also stated that natural mineral waters are not free of bacteria, and counts of 10^4^–10^5^ CFU/mL can be reached within a few days after bottling [9]. According to numerous studies, the microorganisms most frequently found in bottled natural mineral waters are aerobic heterotrophs, *Pseudomonas* spp, *A*. *hydrophila*, total and fecal coliform, *Escherichia coli*, and opportunistic pathogens (Flavobacterium and Mycobacterium) [9, 11, 13, 15–19]. Huguet et al. described that bottled mineral water includes four viruses (Polivio virus 1, Hepatitis A virus, Norovirus, and the Ms 2 phage). Several studies conducted on microbiology of water distribution systems and bottled waters showed fungal growth in water distribution systems and fungi spoilage in bottled mineral waters [20]. 

Mineralized water can also offer a significant amount of required nutrient such as calcium and magnesium [7]. Other components such as carcinogenic compounds, insecticides as DDT, and heavy metals [5], generally, are present in mineralized water in trace concentrations, whose effects on human health are inadequately understood or unknown [7]. Contamination of bottled water with nitrate and nitrite affects the health of infants more than adults, as they are more susceptible to serious health conditions such as Methemoglobinemia, due to the difference in their body size and chemical conditions of the body [8]. Cidu et al. testified an evaluations on the quality of drinking waters. They found that concentrations of Cl^−^, SO_4_^2−^, NO_3_^−^, F^−^, and As^−^ were higher than water quality guidelines [3]. Chloride, sodium, and potassium amount introduced through water is actually very small, and magnesium and calcium content varies remarkably depending on the kind of water [12]. 

In the present study, the chemical and microbiological characteristics of 21 brands of bottled water sold in Isfahan market, one of the most important central province in Iran, were investigated and questioned their accuracy and precision with the levels reported on manufacturer's labeling. Also, the results were compared by the current Codex [21] regulation regarding bottled water in the world. 

## 2. Materials and Methods

### 2.1. Sampling

The 21 analyzed brands of bottled mineral water samples in this research were the most consumable and very high sales brands between other bottled mineral waters in Isfahan, Iran. They were obtained from shops in the city of Isfahan, Iran in 2012. The bottled mineral water samples were opened in the laboratory and analyzed regarding the following anions, fluorides (F^−^), chlorides (Cl^−^), and sulfates (SO_4_^2−^), and cations which include potassium (K^+^), calcium (Ca^2+^), magnesium (Mg^2+^), pH, and bacteriological contaminations for three months.

### 2.2. Materials Used

Only potassium reagent powder pillow was purchased from the Hach company (HACH LANGE, USA) and other materials such as reagents, acid, base, EDTA and used salts BaCl_2_, and NaCl, were purchased from the Merck company (Germany).

### 2.3. Laboratory Analysis

Determination of K^+^ ions was implemented according to Tetra phenyl borat method [23]. The Ca^2+^ and Mg^2+^ ions were analyzed by titration method with EDTA, and for Cl^−^ ions was performed by Mohr method. The SO_4_^2−^ ions were detected by Turbidimetry method, and F^−^ ion was measured by colorimetric method [24]. Microbiological properties of sampled bottled mineral waters were analyzed according to Most Probable Number (MPN/100 mL) multiple-tube fermentation method. Analyses of heterotrophic plate count (HPC) were performed by using of R_2_A agar medium as standard methods [24].

### 2.4. Statistical Test

All the statistical analysis was done with the SPSS statistics package program (Windows version 18.0). The results were stated as mean ±  SD. Comparison of anions and cations concentrations and pH, MPN, and HPC between natural mineral waters and Codex regulations in terms of statistical analyses were directed by student's *t*-test. In all data analysis, a value of *P* < 0.05, *P* < 0.01, and *P* < 0.001 was considered statistically significant.

## 3. Results and Discussion

In this study the amount of pH and ions: F^−^, SO_4_^2−^, Ca^2+^, Mg^2+^, K^+^, and Cl^−^ was surveyed, in 21 samples of bottled mineral waters in Isfahan, Iran. Results showed that there were differences between amounts of these ions in their water contents and label insertion on the bottles (Table 1). Vandevijvere et al. reported that the fluoride levels were different between label and water contents of analyzed bottled mineral waters in Belgium [25]. This result is in opposite of our results that fluoride contents were the same as label values insertion. For magnesium, there was not any value inserted on studied bottled mineral water labels. For potassium and sulfate, respectively, about 43 and 52 percent of studied bottled mineral water contents had values higher than label amounts. Whereas for Ca^2+^, Cl^−^ ions, and pH, our data were about 71, 48, and 67 percent less than label amounts, respectively (Table 1).

Table 2 shows ranges, mean, and standard deviations of results comparing to water quality guidelines. *t*-test indicated that there were significant differences between amounts of each ion by water quality guidelines (*P*_value_<0.001). On the other hand, these differences were not meaningful for potassium ions (K^+^) (*P*_value_>0.05). 

Azlan et al. presented that concentrations of manganese and fluoride in mineral waters were slightly lower than the international standard recommended limits in Malaysia [4]. However in our study, the fluoride results were higher than water quality guidelines. Cidu et al., in 2011, stated that in 20% of the bottled water samples, one or more components have been found at concentrations exceeding the Italian regulations (Cl^−^, SO_4_^2−^, NO_3_^−^, F^−^, and As) [3]. Also, in the present study, concentrations of these ions were exceeded the guidelines which were in opposite of this study results. Cemek et al. indicated that nitrate and nitrite levels in the mineral waters were not found in concentrations considered to be hazardous in terms of public health [5]. Also, Bertoldi et al., based on survey of the chemical composition of 571 European bottled mineral waters, reported that according to European legislation, 9% of samples had boron, nitrate, or nitrite levels above the legal limit existing in individual European countries [10]. Abouleish described that all bottled water samples demonstrated nitrate, nitrite, and other anions levels below the permissible levels accepted by U.S-EPA, U.S-FDA/CFR, and other international organizations, levels exceeding the European commission and drinking water directive (EC/DWD) permissible levels [8], and in our study the mean of all ions except potassium and magnesium had values less than codex guideline values [21]. Chiarenzelli and Pominville showed that except for one sample of mineral water, all bottled waters tested meet United States Environmental Protection Agency (US-EPA) primary standards for drinking water supplies [6].

The total and fecal coliform tests show negative results. In other words, there were not any total and fecal coliforms in these samples. However, the average of heterotrophic plate count (HPC test) in all of the monitored mineral bottled water was 16355 (cfu/mL). Because there are no specific guidelines from WHO for HPC results interpretations in bottled water, the drinking water quality guidelines in distribution systems (500 cfu/mL) were used in comparison to the results [22]. Thus, there were significant differences between obtained results and mentioned guidelines (*P*_value_ < 0.05). 

Falcone-Dias et al. found difference between HPC repeated analyses on R_2_A and on this culture medium supplemented with the beta-lactam amoxicillin. There were insufficient clinical and epidemiological evidence to conclude that the high heterotrophic counts in drinking water pose a risk to consumer's health [9]. Kassenga reported that heterotrophic bacteria were detected in 92% of the bottled mineral waters samples analyzed and total and fecal coliform bacteria were present in 4.6% and 3.6%, respectively, in samples analyzed in Dare-s-Salaam, Tanzania [13], but in our study there were not any total and fecal coliforms. But the heterotrophic bacteria were detected in all 21 sample bottles. Zeenat et al. informed that between 28% and 68% of the samples of the bottled mineral water labels failed to meet the WHO guidelines of 100 CFU/mL and 7% of them also tested positive for fecal coliforms [15]. In the present study, standard of bottled mineral waters was considered 500 CFU/mL [22], thus the 100% of samples which were tested had positive results. 

## 4. Conclusions

In this study, the concentrations of several ions include F^−^, SO_4_^2−^, Ca^2+^, Mg^2+^, K^+^, and Cl^−^ and pH values were measured, in 21 samples of bottled mineral waters in Isfahan, Iran. Then, the sample quality contents were compared with labels on the bottles and then by water quality guidelines. Results showed that there were differences between concentration of these ions and label insertion on the bottles and there were significant differences between concentrations of each ion by water quality guidelines. These differences were not meaningful for potassium ions (K^+^). For other ions these difference were meaningful. Based on microbiological analysis, total and fecal coliforms had negative results. Though, comparison of HPC test with water quality guidelines was meaningful. There was an important total count of these heterotrophic bacteria in the samples. Thus, there is possibility of microorganisms' growth in favorite conditions in bottled water so it was necessary to evaluate the public health risks in bottled water in Iran.

## Figures and Tables

**Table 1:** Comparison of label and real contents of bottled mineral water.

Parameter	% equal to label amounts	% higher than label amounts	% less than label amounts	% not specified in label
K^+^	28.60	42.90	4.80	23.80
Cl^−^	14.30	9.50	47.60	28.60
F^−^	52.40	14.30	0.00	33.33
Ca^2+^	4.80	9.50	71.40	14.30
Mg^2+^	0.00	90.50	0.00	9.50
SO_4_^2−^	14.30	52.40	14.30	19.00
pH	23.80	0.0	66.70	9.50

**Table 2:** Ranges, mean, and standard deviations of results compared to water quality guidelines *.

Parameter	Ranges	Mean ± SD	Bottled mineral water quality guidelines [21]
K^+^	(−0.1)–(4.7)	1.13 ± 1.06 mg/L	0.7 mg/L
Cl^−^	(140.15)–(0)	16.39 ± 31.97 mg/L	400 mg/L
F^−^	(5)–(0)	—	1–1.5
pH	(7.3)–(5.1)	6.60 ± 0.66 mg/L	7.75
Ca^2+^	(49.70)–(11.22)	28.35 ± 10.34	250 mg/L
Mg^2+^	(189.58)–(38.40)	86.58 ± 33.21 mg/L	50 mg/L
SO_4_^2−^	(61.7)–(0.2)	24.17 ± 17.30 mg/L	400 mg/L
HPC	(79667)–(0) cfu/mL	16855.24 ± 25603.213 cfu/mL	500** cfu/mL [22]

*All units are mg/L except HPC that is as cfu/mL.

**Due to lack of bottled mineral water quality guidelines, the drinking water quality guideline was used.
